# On the persistence of supplementary resources in biomedical publications

**DOI:** 10.1186/1471-2105-7-260

**Published:** 2006-05-19

**Authors:** Nicholas R Anderson, Peter Tarczy-Hornoch, Roger E Bumgarner

**Affiliations:** 1 Division of Biomedical Health Informatics, University of Washington, USA; 2 Department of Pediatrics, Department of Computer Science and Engineering, University of Washington, Seattle, Washington, USA; 3 Department of Microbiology, University of Washington, Seattle, Washington, USA

## Abstract

**Background:**

Providing for long-term and consistent public access to scientific data is a growing concern in biomedical research. One aspect of this problem can be demonstrated by evaluating the persistence of supplementary data associated with published biomedical papers.

**Methods:**

We manually evaluated 655 supplementary data links extracted from PubMed abstracts published 1998–2005 (Method 1) as well as a further focused subset of 162 full-text manuscripts published within three representative high-impact biomedical journals between September and December 2004 (Method 2).

**Results:**

For Method 1 we found that since 2001, only 71 – 92% of supplementary data were still accessible via the links provided, with 93% of these inaccessible links occurring where supplementary data was not stored with the publishing journal. Of the manuscripts evaluated in Method 2, we found that only 83% of these links were available approximately a year after publication, with 55% of these inaccessible links were at locations outside the journal of publication.

**Conclusion:**

We conclude that if supplemental data is required to support the publication, journals policies must take-on the responsibility to accept and store such data or require that it be maintained with a credible independent institution or under the terms of a strategic data storage plan specified by the authors. We further recommend that publishers provide automated systems to ensure that supplementary links remain persistent, and that granting bodies such as the NIH develop policies and funding mechanisms to maintain long-term persistent access to these data.

## Background

The large amount of supporting resources necessary to replicate biomedical experiments includes but is not limited to raw data, experimental design specifications, specific software, statistical models, and experimental protocols. Researchers interested in extending or replicating results detailed in a published paper may attempt to use the supplementary resources located at a link within the paper together with their own interpretation of these other factors. Much has been written about the increasingly complex nature of replicating this form of work, from attempts to quantify the ability to replicate the original experimental design, environment, workflow and statistical interpretation. In this paper we focus on the simple ability to retrieve data that original authors felt was of sufficient importance to reference it in support of their results and specifically provide such as supplementary data ostensibly available via an Internet accessible link. While some may question the value or necessity of supplemental data [1] there are numerous reasons for publishing data external to the article itself. These reasons range from size constraints of the journal format and various editorial concerns to the fact that some types of data simply cannot be usefully represented in traditional text or image format. The latter category includes supplemental items such as software (either executable or source code), databases and large data sets that others may wish to re-analyze or include in meta-analyses with other data. Hence, in some cases, supplemental data is a necessity if readers are to evaluate the published work and the persistency of supplemental data is an important concern. To evaluate the long-term availability of supplemental data, we tested for the persistency of the data links from a representative subset of journals indexed within PubMed from 1998 to 2005.

### Data retention and current journal supplementary data policies

Making data freely and easily available should be of concern to most academic researchers who publish in biomedical journals. The National Institutes of Health (NIH) released a Policy Statement in 2003 stating that data must be maintained for three years after the termination of a NIH sponsored grant [2]. In a separate notice from 2002, the NIH also states it "will expect investigators supported by NIH funding to make their research data available to the scientific community for subsequent analyses." [3]. Large research universities are now mandating that research data that is published – whether or not funded by bodies such as the NIH, should be maintained and be easily accessible for up to six years after the conclusion of research as part of their responsible conduct of research policies. It is reasonable to assume that these policies will become more common and widespread in the future. Given that grant funding is generally not available to support long term storage and maintenance of data generated on previous funding and given that researchers may switch institutions, careers or retire, it may not be possible in practice to assure proper data storage and availability for the lengths of time specified by either the NIH or the local institution. Hence, the ability to submit supplemental data to either a journal or a third party data repository, provides a level of stability to data access that may not be achievable by the researcher who is left to his or her own devices.

There is not wide spread agreement between biomedical journals as to consistent supplementary data policies. One reason for this variance is the differing importance and relevance that domain-specific journals place on the different forms of supplementary resources. These resources come in many forms; small and large data sets, experimental protocols, supplementary discussion, links to online biomedical databases, web-based software, source code with or without example data sets, software manuals, etc. Most journals state that data that is directly relevant to a manuscript should be included within the paper, and that additional data that supports conclusions should be made publicly available. Some journals give very specific instructions for each type of desired supplementary resource – manuscripts involving sequences or structural biologic data are typically required to submit the data to a particular public repository prior to or by publication. Other journals state that manuscripts that reference databases should make these databases freely available to all and without password-protection. Few state as clear a policy as the journal *Nature*, which requires that supplementary material need to be stored at either *Nature *or an accredited independent website, and that "such material cannot solely be hosted on an author's personal or institutional site." [4]. *Nature *additionally provides a "Materials complaint" procedure if these guidelines are not followed. In general it appears that supplementary data is accommodated by most biomedical journals, but few appear to require that the data be submitted directly to the journal itself – though this is often possible. However, some journals take an approach that is almost the opposite of Nature and discourage authors from submitting supplemental data but rather suggest that authors host said data on their own site. The apparent motivation for such a policy is to limit the long term cost to journal for data storage and maintenance. Our personal experiences and occasional frustrations in trying to obtain supplemental data led us to perform a study of the persistency of said data.

## Results

### Supplemental data links within PubMed abstracts

**Figure 1:**
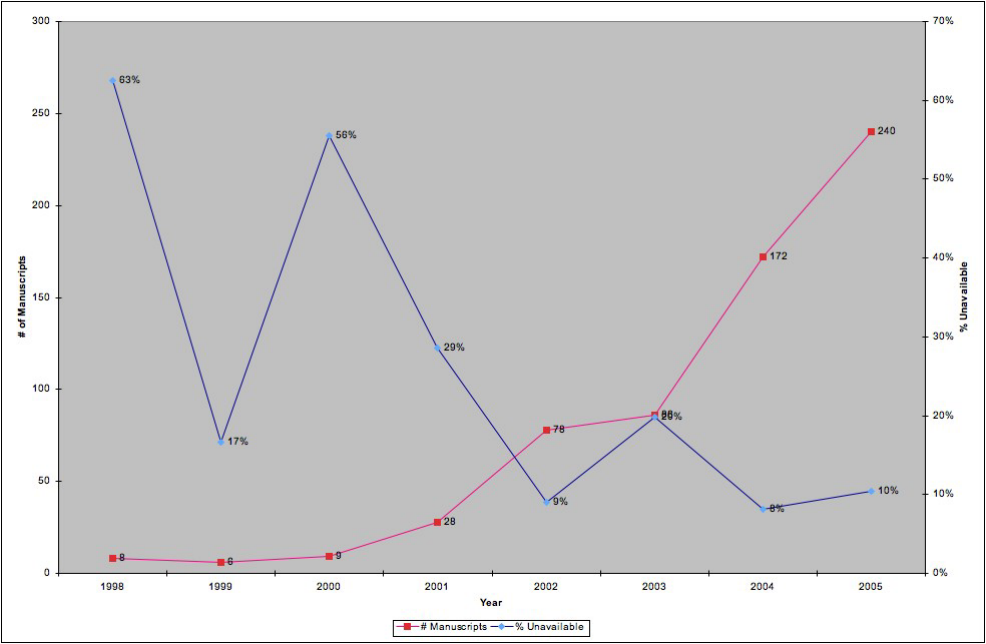
**Number of manuscripts with supplementary links vs. % unavailable**. # Manuscripts vs. % unavailable by year (1998–2005) for method 1.

**Figure 2:**
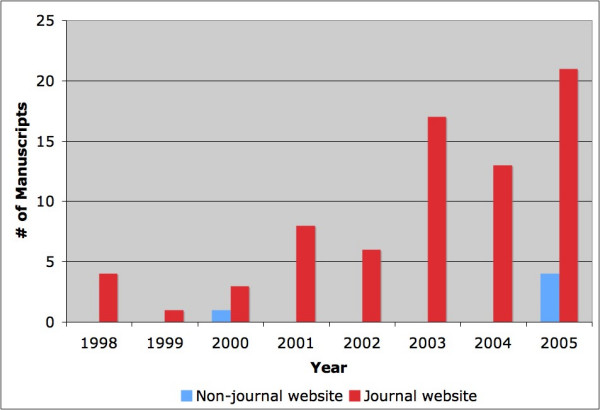
**Location of inaccessible supplementary data**. Journal website vs. non-journal website by year (1998–2005) for method 1.

For the set of records that specified a link within the abstract, we found that an average of 74% of manuscripts published between 1998 and 2005 had links that were still accessible (Chart 1). We note that this result is weighed by the low number of manuscripts published prior to 2001, but still note that an average of 85% of links were still available since 2001. Of the inaccessible links, 93% were to locations outside the journal of publication (Figure 1, 2).

### Supplemental data links within full text manuscripts from three selected journals

Within this set we found an average of 83% of links were available approximately a year after publication. Of the inaccessible links, 55% were to locations outside the journal of publication (Figure 2). This varied between the journals; manuscripts published within *Nucleic Acids *had no links to data outside the journal, whereby *Bioinformatics *had the bulk of its total links to data (73%) referring to locations outside the journal. All of the inaccessible data associated with the journal *Genetics *(33%) came from manuscripts that stated "supplementary data available at genetics.org", where the data was not in fact present at the supplementary data portion of that website (despite reasonable amounts of manual effort to find said data). Despite these individual journal differences and the varying author compliance with supplementary data policies, we feel that the finding of an average of 18% of publications within this dataset having unavailable links confirms the results from 2001–2005 identified via search abstracts alone. In addition, we were quite surprised to find such a large percentage of supplemental data (17%) that was not available only 1 year and a few months past publication. This result combined with a non-zero, recent time, y-intercept on the right of Figure 2, suggests that approximately 10% of all supplemental data links in published articles *never*actually had the supplemental data available. This further suggests that the availability of supplemental data is often not rigorously checked by editors or manuscript reviewers prior to publication.

### Limitations

Our study sampled supplemental data links in both abstracts and a small number of selected full text manuscripts over a 7 year and 3 month time period respectively. Our relatively simple text searches resulted in a fairly small sample size of 655 links from abstracts and 161 links from the selected full text publications. For the earliest years (1998–2000) in which we found links to supplemental data in abstracts, the sample sizes were quite small and it is difficult to draw conclusions from these early data. However, in the later years, a fairly constant 10–20% of the links do not have supplemental data available and these results are consistent with those obtained from our selected full text mining.

It is possible that some of these links we checked were down only temporarily during the time period we checked. Prior work determined that this could be the case up to 19% of the time, but also noted that approximately the same amount (19%) were consistently unavailable [6]. In addition, even if said data was only temporarily missing, it was missing none-the-less and is a reasonable reflection of what a researcher in the field would find. It is also possible that the missing data could have been obtained with further efforts on our part perhaps through direct email contact with authors. However, our goal was not to evaluate whether or not supplemental data could be obtained an any costs but rather to evaluate if data that was ostensibly available through published links could be quickly, easily and conveniently obtained. In addition, it should be noted that automated data mining and aggregation tools would require that such links work.

## Conclusions and recommendations

Biomedical manuscripts are virtually guaranteed to increasingly refer to large data sets and supporting technical material that cannot be contained within the scope of the published manuscript. Journals that are focused on their unique research domains will place different emphasis on the varieties of supplementary data or technical materials relevant to their published manuscripts. A journal interested in public health may, for example, consider data derived from large population based data sets to be crucial to their research, where computational biology journals may place a higher emphasis on software code and example data sets. Despite this variance on the definition of what is relevant, we feel that there is a broad need for improvement in providing persistent access to these resources, regardless of the journal's research focus. There are multiple initiatives at both federal funding agencies and local institutional levels that are calling for greater data sharing and research collaboration. We feel that the following five recommendations address a practical approach to ensuring data persistency for biomedical research publications.

1) Journal policies – At present, journal policies with respect to supplemental data are inconsistent and widely varied with some (such as *Nature*) requiring that all supplemental materials be provided with the manuscript for storage by the publisher or submitted to an independent and credible repository, with other journal policies relatively silent on this issue. Our research shows that supplemental data that is stored on a publisher's website has a significant higher probability of being persistent than data stored on an author's own website. Hence, we encourage all journals to adopt and extend a policy similar to that of *Nature's *if the supplemental data is directly supporting conclusions drawn within the manuscript. This policy states that others should be able to replicate and build upon the author's claims, that specialized data such as DNA sequences or atomic coordinate data must be submitted to and referenced from a third party repository such as PDB/GenBank/EMBL/DDBJ or SWISS-PROT and that an author's own web site is not acceptable for these forms of data. The policy further states that any supporting data sets should be deposited in publicly accessible databases wherever possible, but for occasions for which there is no public repository they should be made available at the authors own website – though this can be cause for refusal of publication if the *Nature *referees cannot be assured of the resources being freely available to the community. Most importantly, the journal also provide a "Materials Complaint Procedure" [9] that allows readers to complain to the journal for problems with gaining access to supplementary data for published manuscripts. At this time *Nature *does not have a recommendation for access to dynamic data such as software source code. If a third party attempting to check or reproduce the results is likely to require or strongly benefit from availability of the supplemental data, we feel the publisher is has an obligation to assure that such data is available for review – either by storing the data on their own site or by requiring the authors to submit it to a credible third party for redistribution. In addition, we encourage publishers to considering accepting and maintaining other supplemental data described within the manuscript that authors feel would benefit the research community even when such data is not directly required to support the conclusions of the manuscript. In many cases, data produced as part of a publication but not specifically required to support the conclusions drawn within, could be useful to others who have different research questions or who wish to mine this data in a larger context.

2) Authors should be required to call out all links within a manuscript either in a specially labelled section near the beginning or end of the manuscript or via separate entry into a web-based form upon submission. The motivation of gathering all links in a common and separate area of the manuscript is to make it simpler for reviewers to identify and check the availability of the resources at said links. In addition, this process (especially if all links were submitted separately in a web based form) would make it easier for automate checking of data availability.

3) Publishers should develop systems to automatically check if links provided within submitted manuscripts are "alive". While this would not assure that the correct data was available at such a link, it would catch the majority of problems we have discovered in which external links are simply not available or in which the provided URL was malformed or mistyped. In addition, we feel it would be wise for publishers to develop a database of all links to supplemental data within manuscripts so that ongoing monitoring of data availability could be accomplished post-publication. Such a system could be developed to contain the original link, the authors' email addresses, a redirected link and perhaps a small amount of associated annotation (a reference to the article and brief text description of the data). With such a system in place, it would be a simple matter to write a script that would perform regular checks to see if the link provided was still available. When it was not, an email notification could be sent to the authors alert them to this fact so that the problem could be corrected or a re-directed link could be provided. While this would not solve all problems associated with missing supplemental data, we posit that a proactive approach such as that suggested would significantly increase overall data availability.

4) Reviewers and editors should be specifically required to assure that all supplemental data is actually available upon submission. Our work suggests that approximately 10% of all supplemental data was not available at the time of publication which further implies the data availability was not carefully checked in the review or editing process.

5) We encourage the NIH to develop not only policies but more importantly, funding mechanisms and/or NIH supported sites for the long term storage and maintenance of heterogeneous supplemental data. We recognize that certain types of supplemental information – such as dynamically generated web sites that are connected to sophisticated databases and/or analytical tools – may necessarily require storage and maintenance by the authors. However, supplemental data that is instantiated in flat files (documents, spreadsheets, images, source code, executable code etc) should be stored in a system designed for long-term data persistency. In addition, we would encourage the NIH to perform an informal audit of the ability of researchers to comply with presently existing policies when funding for long term data storage is not necessarily provided to either the researcher or the researcher's institution. Our own experience, while admittedly anecdotal, suggests that long term maintenance of digital data within a researcher's own lab is often not effectively managed due to a variety of circumstances that include a lack of funding to adequately support an internal IT infrastructure, a lack of sophistication in data storage and backup, and social/human factors.

In conclusion, we feel that long-term persistent access to the rapidly increasing and predominantly digital data that supports modern biomedical research should be treated with the same diligence applied to the published research work itself. Journal publishers are helping drive their individual fields, and as such have a special responsibility to maintain accurate references to supplementary data that specifically supports conclusions in their manuscripts for both present and future researchers. Our work suggests that the assurance of data persistency should not be left solely to the authors, but should be managed by clear policies of the publishing journal or other responsible institution. In addition, while we do not specifically address data persistency for the considerably larger set of data that is not published, our work suggests that the persistency of unpublished data is likely to also become a future research issue. Funding organizations such as the NIH and NSF may need to develop additional policies and more importantly – specific funding mechanisms to assure that such data is available into the future. Similar issues are likely faced by major funding agencies in other countries, so these recommendations may have merit outside the US.

## Methods

We evaluated the persistency of supplementary resources using two complementary methods.

### Method 1

PubMed was searched in January 2006 for abstracts containing the words ("supplementary data" OR "supplemental data" OR "supplementary information" OR "supplementary material") AND ("ftp" OR "www" OR "http" OR "e-mail" OR "email") for each year from 1998 to 2005, which returned 655 unique records. This text was then parsed to isolate links within each abstract, each of which was then checked manually. As detailed in earlier work [5-7], an initial problem is the large # of malformed links within the text. The availability of the data at each of the links (defined as either a URL or FTP address) was manually checked in mid-January 2006.

### Method 2

Since many links to supplemental data are provided in the main body of the text rather than the abstract, it is possible that by focusing only on abstracts we are biasing our sample. Hence, we evaluated a subset of records published over a three-month window approximately one year prior to the present date, where we identified links located within the full text of the publication. We choose three high impact factors (IF) journals from 2004 [8] that had been represented within our initial PubMed data set to evaluate for persistency of supplementary resources: *Bioinformatics *(IF 5.7), *Nucleic Acids Research *(IF 7.26) and *Genetics *(IF 4.1). These journals were chosen for several reasons. First, they all make full text searches of their published manuscripts available through their own web site (for many other journals we would have to download PDFs and search them locally). Second, these journals had relatively high numbers of hits in our searches of abstracts so we could reasonably anticipate that we would find large numbers of supplemental data links within small samples of full text manuscripts. Third, these journals all have reasonably high impact factors and sample a variety of different types of biological researchers (from "bench biologist" to bioinformatician). We searched the full text of all the publications in each journal for manuscripts published between October and December of 2004 for the phrases "supplementary information", "supplemental data", "supplemental material" or "supplementary data". This returned 71, 60 and 30 unique manuscripts for *Bioinformatics*, *Nucleic Acids Research *and *Genetics*, respectively.

For both method 1 and 2, each link was manually checked to determine if the supplemental data mentioned in the text was available [see Additional file 1]. In addition, we each link was categorized by whether the supplemental data was hosted on the journal or a non-journal (typically the authors') website.

## Authors' contributions

NRA participated in the design, performed the experiment and drafted the manuscript, PTH participated in study design and helped draft the manuscript, REB conceived of the study, participated in the design and helped draft the manuscript. All authors have read and approve the final version of this manuscript.

## Supplementary Material

Additional File 1 Supplementary data in the form of an Excel spreadsheet (696 K) detailing the data supporting Method 1 and Method 2 is available online from BMC Bioinformatics. Click here for file

## Figures and Tables

**Figure 3:**
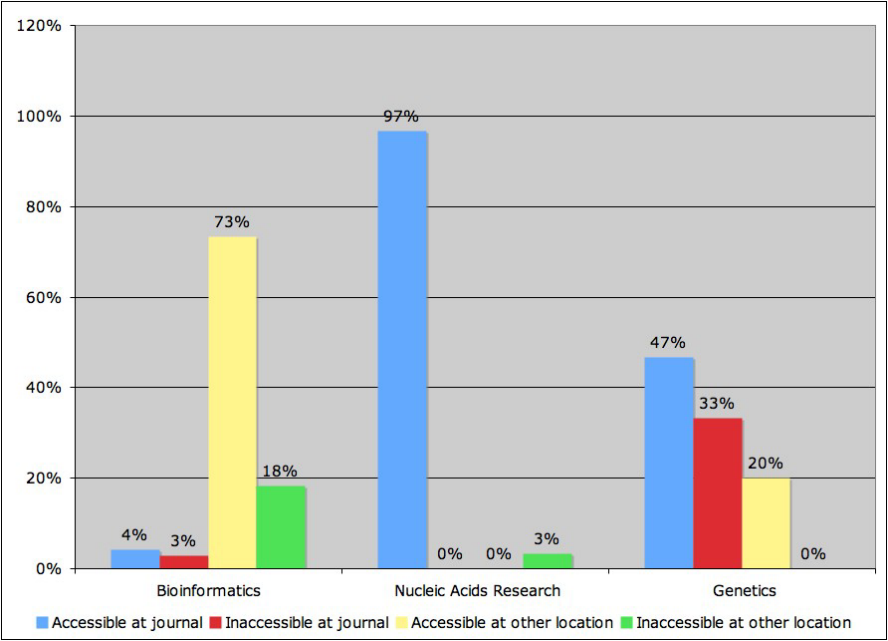
**Accessibility by location of supplementary resources data**. Accessibility at either journal or other location, 9/1/2004–12/31/2004 for method 2.
